# Interactions between Zoliflodacin and *Neisseria gonorrhoeae* Gyrase and Topoisomerase IV: Enzymological Basis for Cellular Targeting

**DOI:** 10.1021/acsinfecdis.4c00438

**Published:** 2024-07-31

**Authors:** Jessica A. Collins, Gregory S. Basarab, Kelly Chibale, Neil Osheroff

**Affiliations:** † Department of Biochemistry, Vanderbilt University School of Medicine, Nashville, Tennessee 37232, United States; ‡ Holistic Drug Discovery and Development (H3D) Centre, University of Cape Town, Rondebosch 7701, South Africa; § Holistic Drug Discovery and Development (H3D) Centre, and South African Medical Research Council Drug Discovery and Development Research Unit, Department of Chemistry and Institute of Infectious Disease and Molecular Medicine, University of Cape Town, Rondebosch 7701, South Africa; ∥ Department of Medicine (Hematology/Oncology), Vanderbilt University School of Medicine, Nashville, Tennessee 37232, United States

**Keywords:** gyrase, topoisomerase IV, spiropyrimidinetrione, zoliflodacin, DNA cleavage, DNA supercoiling/decatenation

## Abstract

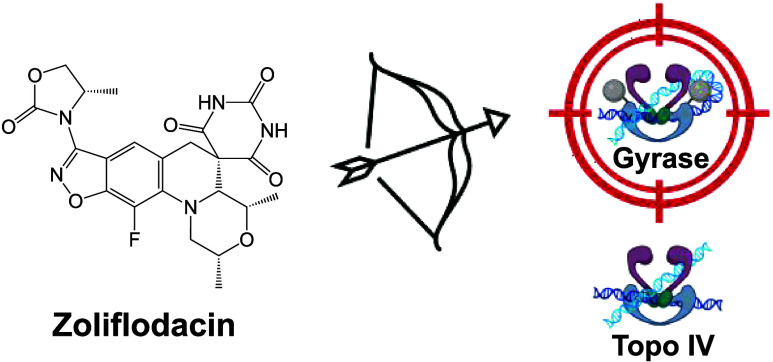

Gyrase and topoisomerase IV are the cellular targets for fluoroquinolones, a critically important class of antibacterial agents used to treat a broad spectrum of human infections. Unfortunately, the clinical efficacy of the fluoroquinolones has been curtailed by the emergence of target-mediated resistance. This is especially true for *Neisseria gonorrhoeae*, the causative pathogen of the sexually transmitted infection gonorrhea. Spiropyrimidinetriones (SPTs), a new class of antibacterials, were developed to combat the growing antibacterial resistance crisis. Zoliflodacin is the most clinically advanced SPT and displays efficacy against uncomplicated urogenital gonorrhea in human trials. Like fluoroquinolones, the primary target of zoliflodacin in *N. gonorrhoeae* is gyrase, and topoisomerase IV is a secondary target. Because unbalanced gyrase/topoisomerase IV targeting has facilitated the evolution of fluoroquinolone-resistant bacteria, it is important to understand the underlying basis for the differential targeting of zoliflodacin in *N. gonorrhoeae*. Therefore, we assessed the effects of this SPT on the catalytic and DNA cleavage activities of *N. gonorrhoeae* gyrase and topoisomerase IV. In all reactions examined, zoliflodacin displayed higher potency against gyrase than topoisomerase IV. Moreover, zoliflodacin generated more DNA cleavage and formed more stable enzyme-cleaved DNA-SPT complexes with gyrase. The SPT also maintained higher activity against fluoroquinolone-resistant gyrase than topoisomerase IV. Finally, when compared to zoliflodacin, the novel SPT H3D-005722 induced more balanced double-stranded DNA cleavage with gyrase and topoisomerase IV from *N. gonorrhoeae*, *Escherichia coli*, and *Bacillus anthracis*. This finding suggests that further development of the SPT class could yield compounds with a more balanced targeting against clinically important bacterial infections.

The bacterial type II topoisomerases are the targets for fluoroquinolones, which are among the most important antibacterials in clinical use.^1−6^ These enzymes, gyrase (GyrA/GyrB) and topoisomerase IV (ParC/ParE), are homologous heterotetrameric enzymes composed of two “A” subunits (GyrA/ParC) and two “B” subunits (GyrB/ParE).^7,8^ The A subunits each contain an active site tyrosine residue that is responsible for cleaving and ligating the DNA substrate.^9−11^ The B subunits each possess a site for the ATP cofactor.^10−12^ The binding and hydrolysis of ATP drive the structural rearrangements in gyrase and topoisomerase IV that are required for overall catalytic activity.^10−13^

Even though gyrase and topoisomerase IV are homologous, they perform different functions in bacterial cells.^8,14,15^ Gyrase primarily modulates the superhelical state of the bacterial chromosome and removes positive supercoils that accumulate ahead of replication forks and transcription complexes.^6,16−19^ Topoisomerase IV primarily untangles catenated daughter chromosomes that are formed during replication.^19−24^ These enzymes use a double-stranded DNA passage reaction to modulate the topological state of DNA and perform their cellular functions.^1,2,6,9,17,25^ During this reaction, an intact double helix is transported through a transient double-stranded break that is created in a separate segment of DNA (i.e., the DNA gate).^1,2,6,9,17,25^ In order to maintain genomic integrity while the DNA gate is open, gyrase and topoisomerase IV form covalent bonds between their active site tyrosine residues and the newly generated 5′–termini of DNA.^1,2,6,17,25^ This covalent enzyme-cleaved DNA complex is known as the cleavage complex.^1,2,6,9,17,25^

Fluoroquinolones exert their antibacterial effects by stabilizing the gyrase/topoisomerase IV cleavage complex.^1,2,6,17^ Unfortunately, for many infections, the increased prevalence of target-mediated resistance has curtailed the medical utility of the fluoroquinolone class.^1−3,6,26^ This resistance is associated with mutations in two highly conserved residues in the catalytic core of gyrase and topoisomerase IV,^1,2,6^ a serine (originally described as Ser83 in the GyrA subunit of *Escherichia coli* gyrase)^27,28^ and an acidic amino acid four residues downstream (Asp87 in *E. coli* GyrA). The corresponding residues in *Neisseria gonorrhoeae* gyrase and topoisomerase IV are GyrA S91/D95 and ParC S87/E91, respectively.

Target-mediated fluoroquinolone resistance has been exacerbated by the fact that members of this drug class do not display balanced targeting between gyrase and topoisomerase IV.^1,2,6^ With rare exceptions,^29−31^ the primary cellular target of fluoroquinolones is gyrase and the secondary target is topoisomerase IV.^1,2,6^ This unbalanced targeting profoundly impacts the development of drug resistance in two ways: a single mutation in gyrase is often adequate (1) to induce sufficient fluoroquinolone resistance to allow bacterial cells to escape drug toxicity, or (2) to allow bacteria to acquire additional mutations in either gyrase or topoisomerase IV and evolve into highly resistant infections.^6,26,32,33^

To address the growing crisis of antibacterial resistance, new classes of clinically advanced compounds have emerged that target gyrase and topoisomerase IV.^6,34−38^ Zoliflodacin and gepotidacin are the leading clinical candidates of the spiropyrimidinetrione (SPT) and triazaacenaphthylene classes, respectively.^39−44^ These compounds interact with the enzymes at amino acid residues that are distinct from those in the fluoroquinolone binding pocket and maintain high activity against fluoroquinolone-resistant bacterial strains.^39,45−52^

Both zoliflodacin and gepotidacin display notable activity against gonorrhea.^39,45−48,50,53^ Results from phase III clinical trials that examined the use of zoliflodacin or gepotidacin for the treatment of uncomplicated urogenital gonorrhea report positive outcomes.^41,43^ Gonorrhea is a sexually transmitted infection that is a global concern with more than 82 million new cases occurring worldwide in 2022.^54^ The etiological agent of gonorrhea is the Gram-negative diplococci *N. gonorrhoeae*,^55^ which infects the mucosal epithelium of the genitals, rectum, and throat.^56^ Untreated gonorrheal infections can cause severe complications, including pelvic inflammatory disease, infertility, and when disseminated, endocarditis and bacteremia.^55,56^ The fluoroquinolone ciprofloxacin was frontline therapy for gonorrhea until 2006 when it was removed from treatment guidelines by the Centers for Disease Control and Prevention due to high levels of target-mediated resistance.^57^

In contrast to gepotidacin, which displays well-balanced dual targeting in multiple species,^6,50,52,58,59^ microbiological studies indicate that zoliflodacin, like fluoroquinolones,^1,2,6^ displays unbalanced targeting of the type II topoisomerases in *N. gonorrhoeae* with gyrase being the primary cytotoxic target.^39,46,60^ Given the clinical promise of zoliflodacin and the fact that unbalanced targeting can potentiate target-mediated resistance, it is important to understand the underlying basis for the differential targeting of zoliflodacin in *N. gonorrhoeae*. Therefore, we assessed the effects of this SPT on the catalytic and DNA cleavage activities of *N. gonorrhoeae* gyrase and topoisomerase IV.

In all reactions examined, zoliflodacin displayed higher potency against gyrase than topoisomerase IV. Furthermore, zoliflodacin enhanced DNA cleavage to a greater extent and formed more stable enzyme-cleaved DNA-SPT complexes with gyrase over topoisomerase IV. In addition, the SPT maintained activity against fluoroquinolone-resistant gyrase better than it did against resistant topoisomerase IV. Finally, H3D-005722, a novel member of the SPT class,^61^ displayed more balanced activity against gyrase and topoisomerase IV from *N. gonorrhoeae*, *E. coli*, and *Bacillus anthracis* compared with zoliflodacin in double-stranded DNA cleavage assays. This last finding suggests that further development of the SPT class could yield compounds with better balanced targeting against bacterial pathogens that impact human health.

## Results

SPTs, like fluoroquinolones, interact with the cleavage complex and have two effects on gyrase/topoisomerase IV activity.^6,49,62−64^ First, they stabilize the cleavage complex, leading to higher equilibrium concentrations of enzyme-generated DNA breaks.^1−3,6,26,33,46,62^ Two molecules of fluoroquinolones and SPTs interact with the enzymes (one inserting at each scissile bond).^49,62−64^ Consequently, these drugs primarily enhance gyrase/topoisomerase IV-mediated double-stranded breaks.^6,46,65^ Second, because fluoroquinolones and SPTs stabilize cleavage complexes, they impair the ability of gyrase and topoisomerase IV to proceed through their catalytic cycles.^2,6,17,26,46,66^ As a result, these drugs also inhibit the overall catalytic activities of the two enzymes.^6,46,61,65^

Gyrase is the primary cellular target for zoliflodacin in *N. gonorrhoeae*.^60^ Kern et al. showed that the SPT inhibits the catalytic activities of *N. gonorrhoeae* gyrase and topoisomerase IV and increased levels of double-stranded DNA breaks with both enzymes.^46^ However, these studies did not offer insight into the differential targeting of zoliflodacin for gyrase over topoisomerase IV. Therefore, as a first step to address this important issue, we revisited the ability of zoliflodacin to inhibit overall catalytic activity and enhance DNA cleavage mediated by *N. gonorrhoeae* gyrase and topoisomerase IV.

### Effects of Zoliflodacin on Catalysis and DNA Cleavage Mediated by *N. gonorrhoeae* Gyrase and Topoisomerase IV

**Figure 1:**
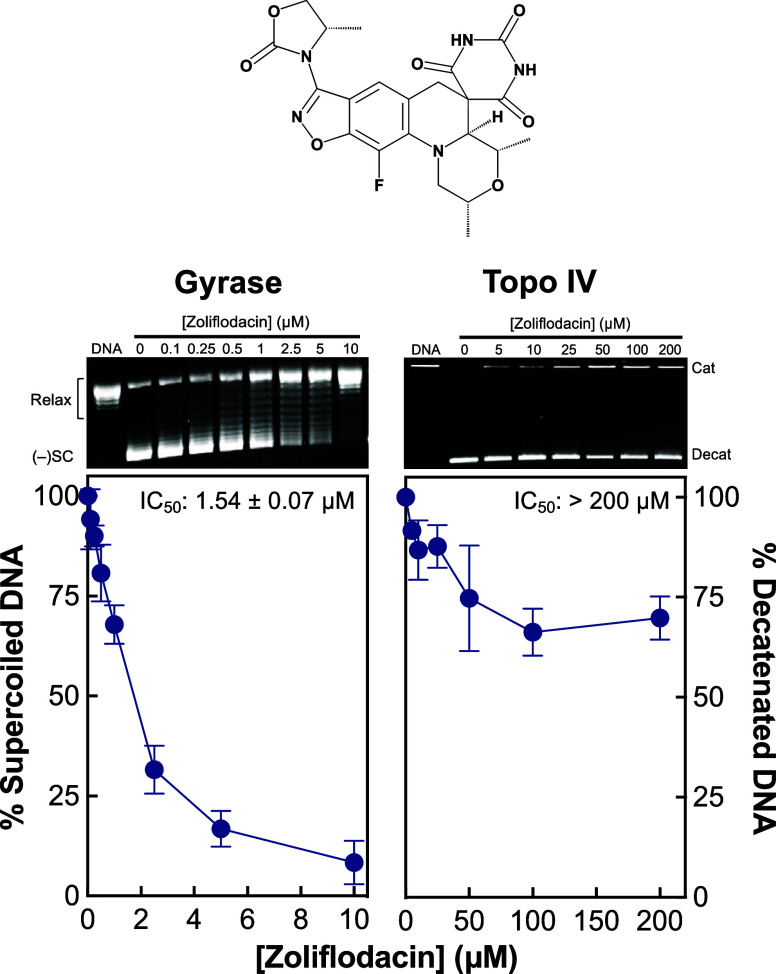
Zoliflodacin inhibits the catalytic activities of *N. gonorrhoeae* gyrase and topoisomerase IV. The ability of zoliflodacin to inhibit gyrase-catalyzed DNA supercoiling (left panel) and topoisomerase IV-catalyzed DNA decatenation (right panel) is shown. Error bars represent the standard deviation of at least 3 independent experiments. IC_50_ values (drug concentration at which enzyme activity is inhibited by 50%), including the standard error of the mean, for each assay are shown in the respective panels. Representative gels of gyrase-catalyzed DNA supercoiling (left) and topoisomerase IV-catalyzed DNA decatenation (right) with zoliflodacin are shown above the graphs. DNA represents the fully relaxed (left) or fully catenated (right) DNA control. The positions of relaxed (Relax), negatively supercoiled [(−)SC], catenated (Cat), and decatenated (Decat) plasmids are indicated. The structure of zoliflodacin is shown at the top.

Figure 1 shows the inhibition of gyrase-catalyzed DNA supercoiling (left panel) and topoisomerase IV-catalyzed DNA decatenation (right panel) by zoliflodacin. The SPT was a potent inhibitor of DNA supercoiling with an IC_50_ value of ∼1.7 μM. This value is ∼4-fold lower than reported previously.^46^ Although the previous study reported an IC_50_ value for decatenation that was ∼3-fold higher than that for supercoiling,^46^ we found a far larger difference. Even at zoliflodacin concentrations as high as 200 μM, we never observed the DNA decatenation activity of topoisomerase IV drop below 50%.

**Figure 2:**
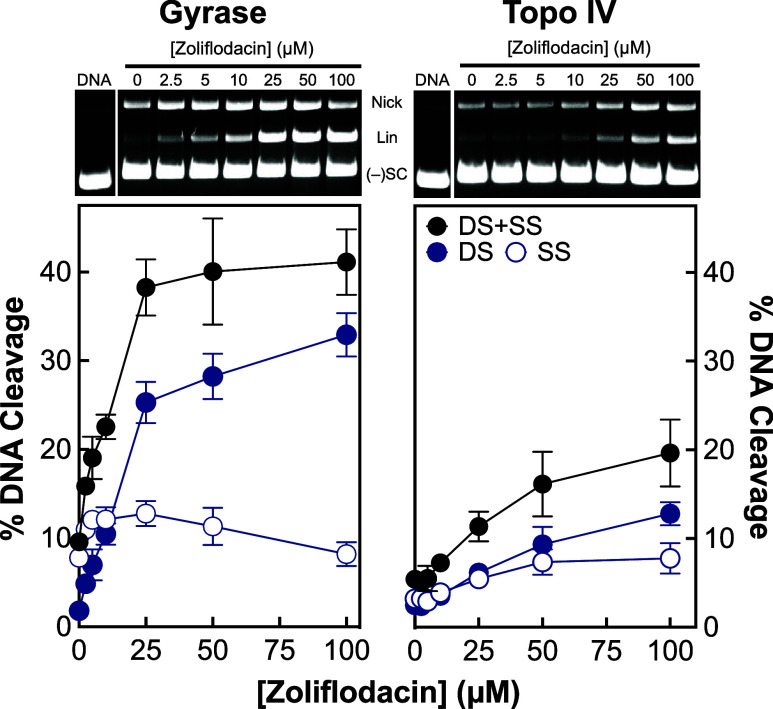
Zoliflodacin enhances double-stranded and single-stranded DNA scission mediated by *N. gonorrhoeae* gyrase and topoisomerase IV. The ability of zoliflodacin to induce double-stranded (DS, blue, closed circle), single-stranded (SS, blue, open circle), and total (black, DS + SS, closed circle) DNA cleavage mediated by gyrase (left panel) and topoisomerase IV (right panel) are displayed. Error bars represent the standard deviation of at least 3 independent experiments. Representative gels of zoliflodacin-induced DNA cleavage mediated by gyrase (left) and topoisomerase IV (right) are shown at the top. DNA represents the negatively supercoiled DNA control. The positions of nicked (Nick), linear (Lin), and negatively supercoiled [(−)SC] plasmid are indicated. Note that the DNA control lane (DNA) was located on the same gels but several lanes away from the 0 μM zoliflodacin lane. Intermediary lanes, which included a compound that was not relevant to the present study, were removed for clarity.

To further evaluate the effects of zoliflodacin on *N. gonorrhoeae* gyrase and topoisomerase IV, we assessed the ability of the SPT to enhance DNA cleavage mediated by both enzymes (Figure 2). Zoliflodacin was ∼3.4-fold more potent against gyrase-mediated (left panel) compared with topoisomerase IV-mediated (right panel) double-stranded DNA cleavage (on the basis of the zoliflodacin concentration required to increase double-stranded DNA cleavage to 50% maximal levels, ∼16.2 and ∼55.0 μM for gyrase and topoisomerase IV, respectively). Once again, differences in the potency of zoliflodacin against gyrase and topoisomerase IV were greater than those described by Kern et al.^46^

Other factors being equal, the antibacterial activity of fluoroquinolones (and other gyrase/topoisomerase IV-targeted drugs that induce DNA cleavage) correlates with increased concentrations of enzyme-DNA cleavage complexes.^6,26^ Unfortunately, the previous study did not report maximal levels of DNA scission induced by zoliflodacin with the *N. gonorrhoeae* type II topoisomerases.^46^ Therefore, we quantified levels of gyrase- and topoisomerase IV-mediated DNA cleavage generated in the presence of zoliflodacin. As seen in Figure 2, the SPT induced considerably higher levels of double-stranded DNA cleavage with gyrase than topoisomerase IV (32.9 vs 12.0% at 100 μM).

In addition to double-stranded DNA breaks, zoliflodacin also induced enzyme-mediated single-stranded DNA breaks. As evidenced by studies with novel bacterial topoisomerase inhibitors (NBTIs) and triazaacenaphthylenes, which primarily induce single-stranded rather than double-stranded DNA breaks, gyrase/topoisomerase IV-mediated single-stranded DNA breaks are also lethal to cells.^6,52,67^ When single-stranded breaks are also considered, the total levels of DNA cleavage rise to 41.1% with gyrase and 19.6% with topoisomerase IV at 100 μM zoliflodacin.

Taken together, the above results are consistent with gyrase being the primary cellular target for zoliflodacin in *N. gonorrhoeae* cells; the SPT displayed a greater potency toward gyrase and induced higher levels of DNA cleavage with gyrase compared with topoisomerase IV.

**Figure 3:**
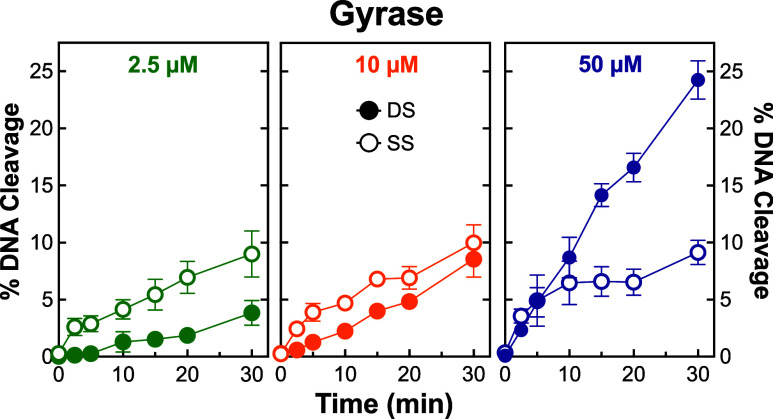
Time courses for DNA cleavage mediated by *N. gonorrhoeae* gyrase. Time courses monitoring levels of double-stranded (DS, closed circle) and single-stranded (SS, open circle) DNA breaks mediated by gyrase in the presence of 2.5 μM (green, left panel), 10 μM (orange, middle panel), and 50 μM (purple, right panel) are shown. Error bars represent the standard deviation of at least 3 independent experiments.

It is notable that levels of zoliflodacin-induced double-stranded and single-stranded DNA breaks increase coordinately with topoisomerase IV (Figure 2, right panel). This finding suggests that the first and second SPT molecules bind to the cleavage complex with similar affinities. In contrast, levels of zoliflodacin-induced single-stranded DNA breaks rise before double-stranded breaks with gyrase, implying that the first zoliflodacin molecule binds to the cleavage complex with a higher affinity than that of the second for this enzyme. To further evaluate the potential sequential binding of zoliflodacin to gyrase, time courses for DNA cleavage were carried out at 2.5, 10, and 50 μM zoliflodacin, concentrations at which single-stranded breaks exceeded, were comparable to, and were lower than double-stranded breaks, respectively (Figure 3). In all cases, the velocity for the formation of single-stranded breaks over 30 min was greater than that for double-stranded breaks, supporting the idea that the two molecules of zoliflodacin bind sequentially to *N. gonorrhoeae* gyrase. This finding further suggests that at low concentrations of zoliflodacin in *N. gonorrhoeae* cells, the SPT may be inducing primarily single-stranded breaks with gyrase.

### Stability of Gyrase/Topoisomerase IV-Mediated Double-Stranded DNA Breaks Induced by Zoliflodacin

**Figure 4:**
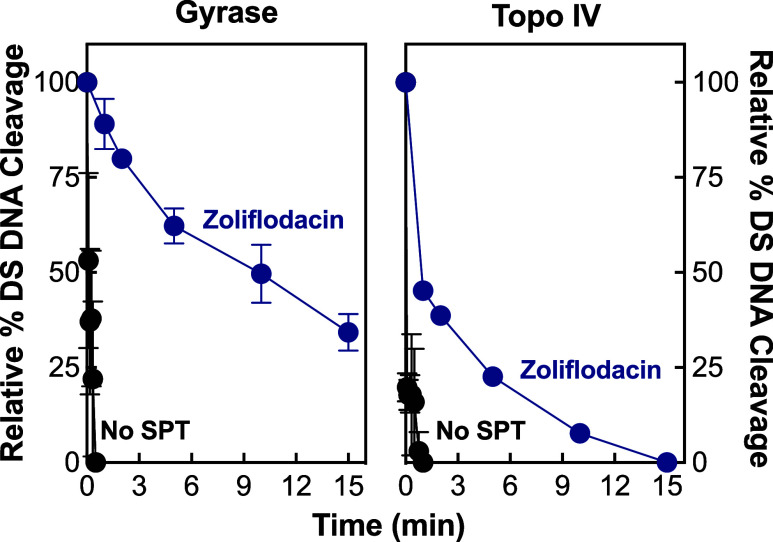
Zoliflodacin induces more stable enzyme-cleaved DNA complexes with *N. gonorrhoeae* gyrase than with topoisomerase IV. Persistence reactions were permitted to reach cleavage–ligation equilibrium prior to 20-fold dilution in reaction buffer lacking MgCl_2_. The stability of the diluted complexes was determined by monitoring the decay of the linear DNA band. Persistence of double-stranded (DS) DNA cleavage complexes generated by gyrase (left panel) and topoisomerase IV (right panel) in the presence (blue) or absence (black, no SPT) of 100 μM zoliflodacin is shown. Levels of DNA cleavage prior to dilution of cleavage complexes were set to 100%. Error bars represent the standard deviation of at least 3 independent experiments.

A study conducted with human type II topoisomerases found that other things being equal, drugs that generate the most stable cleavage complexes are the most cytotoxic to cells.^68^ Therefore, two approaches were employed to investigate the effects of zoliflodacin on the stability of cleavage complexes formed by *N. gonorrhoeae* gyrase and topoisomerase IV. First, the persistence of cleavage complexes in the absence and presence of zoliflodacin was assessed. In the assay, cleavage complexes are formed in the presence of high concentrations of gyrase/topoisomerase IV and DNA, and the lifetimes of cleavage complexes are monitored following a 20-fold dilution into a reaction buffer that lacks the catalytic divalent metal ion. Although the shift in condition does not alter the DNA cleavage–ligation equilibrium in established cleavage complexes, enzyme-DNA-SPT complexes that disassociate are unlikely to reform. As seen in Figure 4, gyrase/topoisomerase IV-DNA cleavage complexes were highly unstable in the absence of drug and rapidly disassociated following dilution (*t*_1/2_ < 5 s). The stability of these complexes increased considerably in the presence of zoliflodacin. Moreover, the gyrase-DNA-SPT complex was ∼10 times more stable (*t*_1/2_ ≈ 10 min, left panel) than the topoisomerase IV complex (*t*_1/2_ ≈ 1 min, right panel).

**Figure 5:**
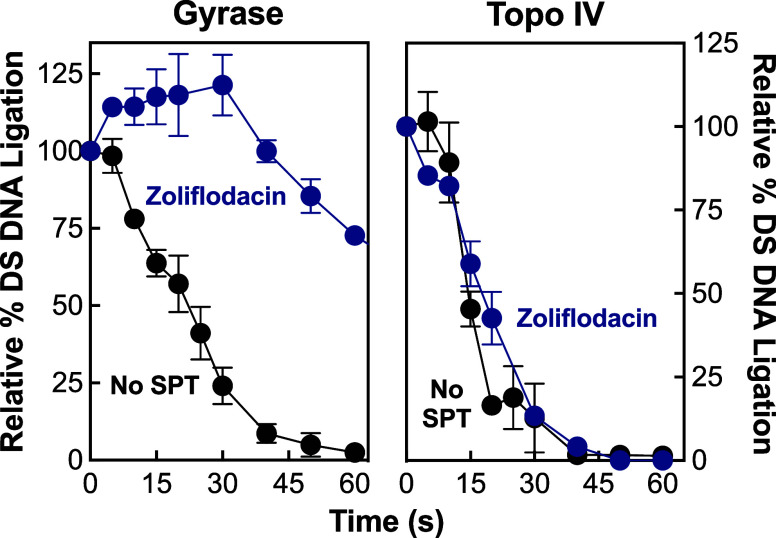
Zoliflodacin is a more potent inhibitor of DNA ligation mediated by *N. gonorrhoeae* gyrase than topoisomerase IV. Ligation of double-stranded (DS) DNA cleavage complexes by gyrase (left panel) and topoisomerase IV (right panel) formed in the presence (blue) or absence (black, no SPT) of 100 μM zoliflodacin are shown. Levels of DNA scission prior to the induction of DNA ligation were set to 100%. Error bars represent the standard deviation of at least 3 independent experiments.

In the second approach, the effects of zoliflodacin on the rates of *N. gonorrhoeae* gyrase/topoisomerase IV-mediated DNA ligation were monitored. In the assay, cleavage complexes are shifted from 37 to 65 °C (a temperature that allows DNA ligation but not DNA cleavage),^69^ and the loss of double-stranded DNA breaks is followed. As seen in Figure 5, cleavage complexes formed in the absence of the SPT were rapidly religated by gyrase (*t*_1/2_ ≈ 19.8 s, left panel) and topoisomerase IV (*t*_1/2_ ≈ 15 s, right panel). Although zoliflodacin had a significant effect on ligation rates for double-stranded DNA breaks with gyrase (*t*_1/2_ > 60 s, left panel), the SPT had little effect on the rates of topoisomerase IV-mediated ligation (*t*_1/2_ ≈ 17 s, right panel). Taken together, these findings indicate that the gyrase-DNA-SPT complex is more stable than the analogous topoisomerase IV complex, which further supports the demonstration that gyrase is the primary cytotoxic target of zoliflodacin in *N. gonorrhoeae*.^45,60^

### Effects of DNA Supercoil Handedness on DNA Cleavage Induced by Zoliflodacin

Previous studies with the type II topoisomerases from *B. anthracis*, *E. coli*, *Mycobacterium tuberculosis*, and *Staphylococcus aureus* indicate that gyrase maintains lower levels of DNA cleavage complexes with positively over negatively supercoiled DNA in the presence of antibacterials.^51,70−72^ Although this property makes gyrase a safer enzyme to function ahead of replication forks and transcription complexes (because DNA strand breaks formed ahead of DNA tracking machinery are more lethal to cells), it potentially diminishes the effects of antibacterials on the generation of cleavage complexes.^1,2,6^ Conversely, studies with *B. anthracis* and *E. coli* topoisomerase IV indicate that levels of cleavage complexes formed with positively and negatively supercoiled DNA are similar.^70,72^ This lack of topology discrimination likely has less impact on the lethality of topoisomerase IV-induced DNA strand breaks, as these are generated on positively supercoiled precatenanes that are formed behind replication forks (where they are protected from approaching DNA tracking machinery that could create chromosome breaks that must be repaired by recombination processes).^1,2,6^

**Figure 6:**
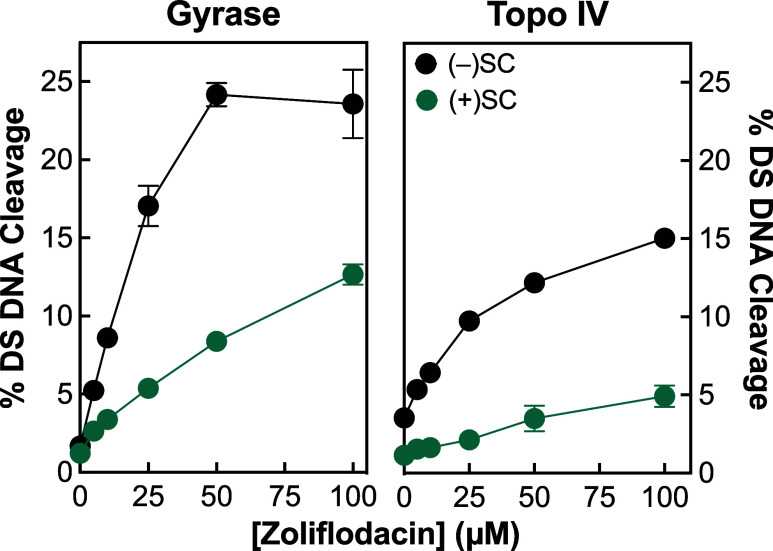
*N. gonorrhoeae* gyrase and topoisomerase IV maintain lower levels of cleavage complexes on positively supercoiled DNA. Levels of cleavage complexes generated by gyrase (left panel) and topoisomerase IV (right panel) with positively supercoiled [green, (+)SC] or negatively supercoiled [black, (−)SC] DNA in the presence of zoliflodacin are shown. Error bars represent the standard error of the mean of at least 2 independent experiments. Levels of topoisomerase IV were increased 2-fold to improve visualization of DNA breaks induced with positively supercoiled DNA.

The effects of supercoil handedness on DNA cleavage mediated by *N. gonorrhoeae* gyrase and topoisomerase IV are shown in Figure 6. (Note that levels of topoisomerase IV were increased 2-fold in these experiments as compared with Figure 2 to improve visualization of DNA breaks induced with positively supercoiled DNA.) Gyrase maintained ∼2- to 3-fold lower levels of zoliflodacin-induced double-stranded DNA breaks with positively compared to negatively supercoiled DNA substrates (left panel). This finding is consistent with previous reports with gyrase from other species. Surprisingly, and in contrast to results from earlier studies,^70,72^ topoisomerase IV also maintained ∼3-fold lower levels of cleavage complexes in the absence and presence of zoliflodacin (right panel). The reduced DNA cleavage activity of topoisomerase IV on positively supercoiled DNA could further diminish its role as a cytotoxic target for zoliflodacin in *N. gonorrhoeae* cells.

### Effects of Fluoroquinolone Resistance Mutations on the Susceptibility of Gyrase and Topoisomerase IV to Zoliflodacin

The effects of fluoroquinolone resistance mutations on the sensitivity of gyrase to zoliflodacin have been examined in two species, *M. tuberculosis* and *N. gonorrhoeae*.^46,73^ Remarkably, the presence of single-resistance mutations at GyrA Ala90 and Asp94 (corresponding to GyrA Ser91 and Asp95 in *N. gonorrhoeae*, respectively) enhanced the ability of zoliflodacin and other SPTs to induce gyrase-mediated DNA cleavage.^73^ Maximal levels of zoliflodacin-induced double-stranded DNA scission mediated by the fluoroquinolone-resistant gyrases were ∼2- to 5-fold higher than those observed with the wild-type enzyme.^73^

In contrast to the study with *M. tuberculosis* gyrase (which utilized single fluoroquinolone resistance mutants),^73^ Kern et al. examined the activity of zoliflodacin against *N. gonorrhoeae* gyrase carrying mutations at both the serine and acidic amino acid residues (GyrA^S91F/D95G^).^46^ In this case, the presence of the two mutations minimally affected the potency of zoliflodacin against the DNA supercoiling and cleavage reactions of *N. gonorrhoeae* gyrase. (Levels of DNA scission were not reported in this study).^46^

**Figure 7:**
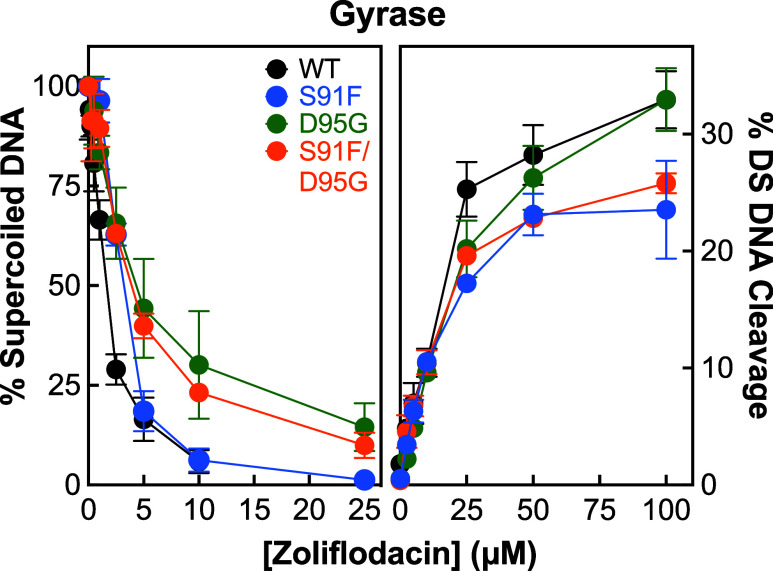
Effects of zoliflodacin on the catalytic and DNA cleavage activities of *N. gonorrhoeae* gyrase harboring fluoroquinolone resistance mutations. The abilities of wild-type (black), GyrA^S91F^ (S91F, blue), GyrA^D95G^ (D95G, green), and GyrA^S91F/D95G^ (S91F/D95G, orange) gyrase to supercoil relaxed plasmid (left panel) or to enhance double-stranded (DS) DNA cleavage (right panel) in the presence of zoliflodacin are shown. Error bars represent the standard deviation of at least 3 independent experiments.

Therefore, to determine whether zoliflodacin might show enhanced activity against the single fluoroquinolone-resistant mutants, we compared the effects of the SPT on DNA supercoiling and cleavage with *N. gonorrhoeae* gyrase harboring GyrA^S91F^, GyrA^D95G^, or GyrA^S91F/D95G^ to those with the wild-type enzyme (Figure 7). Only a slight decrease in zoliflodacin activity was seen with all three mutant enzymes. IC_50_ values for DNA supercoiling catalyzed by mutant gyrase were increased ∼2- to 3-fold (IC_50_ ∼ 3.0, ∼4.4, and ∼3.8 μM with GyrA^S91F^; GyrA^D95G^, and GyrA^S91F/D95G^, respectively) compared to wild-type (left panel, IC_50_ ∼ 1.5 μM). Furthermore, zoliflodacin maintained its potency in DNA cleavage assays, and levels of double-stranded DNA cleavage at 100 μM zoliflodacin dropped at most 9.4% (from 32.9% for wild-type to 23.5% for GyrA^S91F^). These findings are consistent with reports that the susceptibility of *N. gonorrhoeae* cells carrying the GyrA^S91F/D95G^ fluoroquinolone-resistant gyrase is similar to that of wild-type cells.^39,46,47^

**Figure 8:**
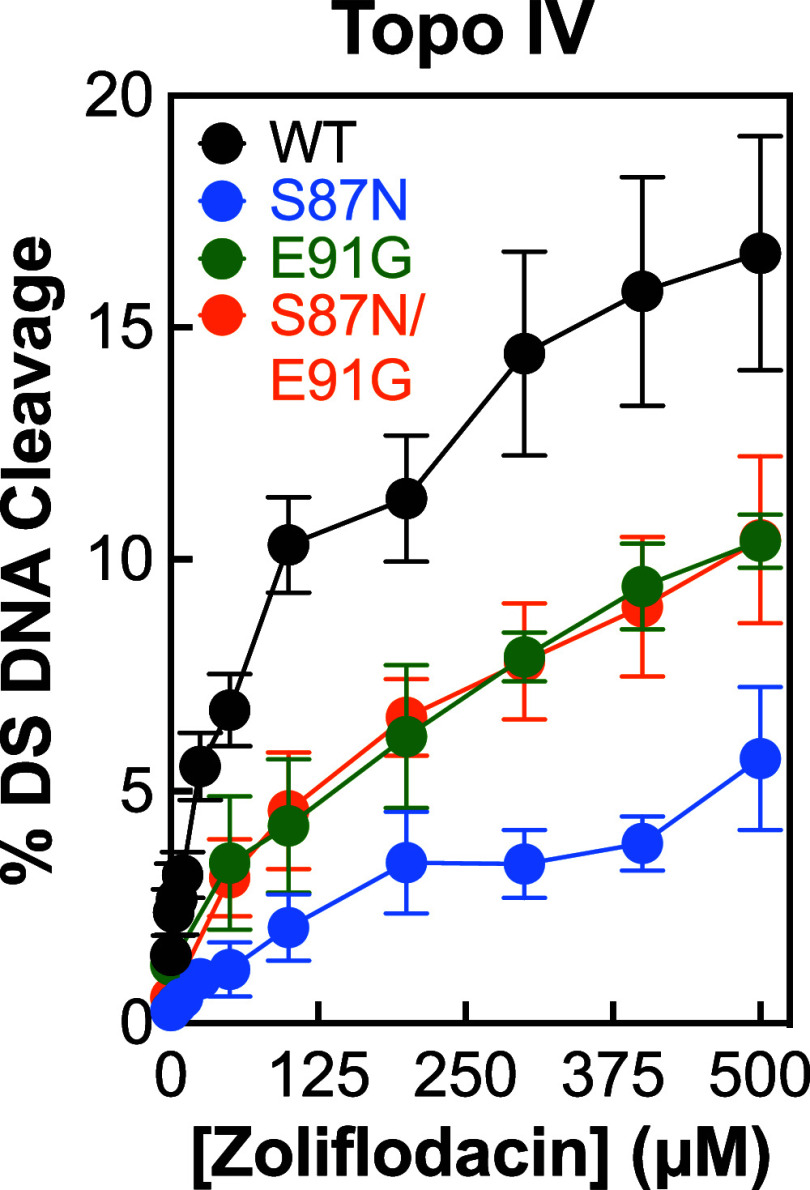
Effects of zoliflodacin on the DNA cleavage activities of *N. gonorrhoeae* topoisomerase IV carrying fluoroquinolone resistance mutations. The ability of zoliflodacin to induce double-stranded (DS) DNA cleavage mediated by wild-type (black), ParC^S87N^ (S87N, blue), ParC^E91G^ (E91G, green), and ParC^S87N/E91G^ (S87N/E91G, orange) topoisomerase IV is shown. Error bars represent the standard deviation of at least 3 independent experiments.

Laboratory and clinical studies have demonstrated that *N. gonorrhoeae* cells carrying mutations in both gyrase and topoisomerase IV display higher levels of fluoroquinolone resistance than those that only encode mutations in gyrase.^74−79^ However, the effects of fluoroquinolone resistance mutations in topoisomerase IV have never been examined with any SPT. Thus, the activity of zoliflodacin against topoisomerase IV carrying ParC^S87N^, ParC^E91G^, or ParC^S87N/E91G^ was evaluated (Figure 8). Because the SPT displayed so little inhibition of decatenation with the wild-type enzyme (Figure 1), our studies focused solely on topoisomerase IV-mediated double-stranded DNA cleavage.

The fluoroquinolone resistance mutations in topoisomerase IV had an obvious effect on the activity of zoliflodacin, with levels of double-stranded DNA scission being reduced by ∼1.6- to 3-fold (at 100 μM SPT). It is not clear whether this diminished activity would affect the susceptibility of cells harboring fluoroquinolone resistance mutations in both gyrase and topoisomerase IV to SPTs. However, because gyrase is the primary cellular target of zoliflodacin in *N. gonorrhoeae* cells,^60^ it is likely that the SPT would maintain its activity against these fluoroquinolone-resistant infections.

### Effects of SPTs against Gyrase and Topoisomerase IV across Species

**Figure 9:**
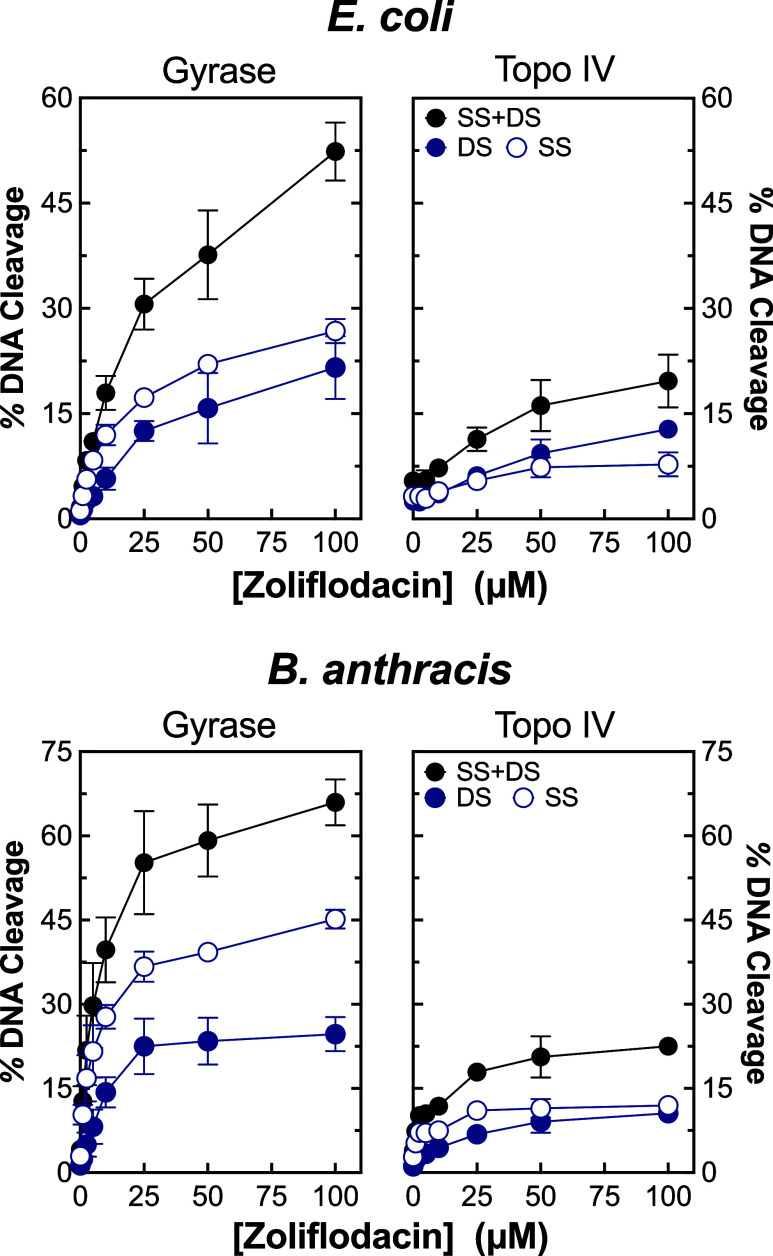
Zoliflodacin enhances DNA cleavage mediated by *E. coli* and *B. anthracis* gyrase and topoisomerase IV. The ability of zoliflodacin to induce double-stranded (DS, blue, closed circle), single-stranded (SS, blue, open circle), and total (black, DS + SS, closed circle) DNA cleavage mediated by gyrase (left panels) and topoisomerase IV (right panels) from *E. coli* (top) and *B. anthracis* (bottom) are shown. Error bars represent the standard deviation of at least 3 independent experiments.

The effects of zoliflodacin on DNA cleavage mediated by type II topoisomerases have been reported only for *M. tuberculosis* and *N. gonorrhoeae*.^46,73^ Thus, to determine whether results can be generalized to other bacterial species, we examined the activity of the SPT on gyrase/topoisomerase IV-mediated DNA scission from *E. coli* and *B. anthracis* (Figure 9). With both species, zoliflodacin generated ∼2 times more double-stranded DNA breaks with gyrase (left panels) than with topoisomerase IV (right panels). Thus, with *E. coli* and *B. anthracis*, the SPT maintains its preference for gyrase at the enzymological level.

In addition, zoliflodacin induced considerably higher levels of single-stranded DNA breaks with gyrase compared with topoisomerase IV from *E. coli* and *B. anthracis*. In fact, zoliflodacin generated even more gyrase-mediated single-stranded than double-stranded DNA breaks at all concentrations examined. As a result, zoliflodacin generated ∼2.5 to 3 times higher levels of total DNA breaks with gyrase than topoisomerase IV from these two species. Therefore, we predict that gyrase should also be the primary cellular target for zoliflodacin in *E. coli* and *B. anthracis* cells.

**Figure 10:**
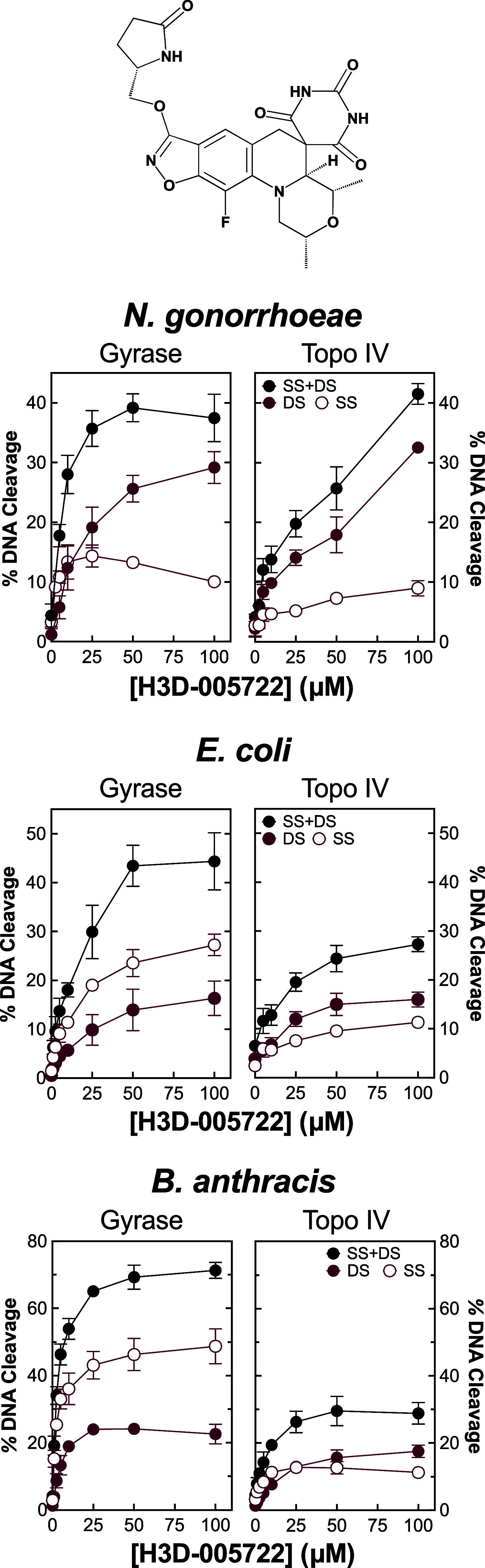
SPT H3D-005722 enhances DNA cleavage mediated by gyrase and topoisomerase IV from *N. gonorrhoeae*, *E. coli*, and *B. anthracis*. The ability of H3D-005722 to increase levels of double-stranded (DS, maroon, closed circle), single-stranded (SS, maroon, open circle), and total (black, DS + SS, closed circle) DNA breaks mediated by gyrase (left panels) and topoisomerase IV (right panels) from *N. gonorrhoeae* (top), *E. coli* (middle), and *B. anthracis* (bottom) is shown. Error bars represent the standard deviation of at least 3 independent experiments. The structure of H3D-005722 is displayed at the top.

Other than zoliflodacin, very little is known regarding the activities of other SPTs against gyrase and topoisomerase IV.^46,73,80^ H3D-005722 (see the inset Figure 10, top, for structure) is an analogue of zoliflodacin in which the oxazolidinone group is replaced by a pyrrolidinone moiety.^61^ This SPT was chosen for comparison because it induces higher levels of DNA cleavage mediated by *M. tuberculosis* gyrase than zoliflodacin.^73^ Consequently, to determine whether the properties of zoliflodacin translate to other members of the SPT class, the effects of H3D-005722 on DNA cleavage mediated by *N. gonorrhoeae*, *E. coli*, and *B. anthracis* gyrase and topoisomerase IV were determined (Figure 10).

The DNA cleavage profile of H3D-005722 with *N. gonorrhoeae* gyrase is very similar to that of zoliflodacin (top left panel). However, the novel SPT was considerably more active against *N. gonorrhoeae* topoisomerase IV (top right panel). To this point, at 100 μM H3D-005722, comparable levels of double-stranded and total DNA strand breaks were observed with gyrase and topoisomerase IV.

In further contrast to zoliflodacin, H3D-005722 generated similar levels of double-stranded DNA breaks with gyrase and topoisomerase IV from *E. coli* (middle panel) and *B. anthracis* (bottom panel). However, as was seen with zoliflodacin in Figure 9, H3D-005722 induced higher levels of single-stranded than double-stranded DNA breaks with gyrase from these two species. Once again, this resulted in higher levels of total DNA breaks mediated by gyrase over topoisomerase IV from *E. coli* and *B. anthracis*.

## Discussion

Zoliflodacin is an advanced clinical stage SPT antibacterial with activity against gyrase and topoisomerase IV.^39−41,46^ The present work provides considerable data that helps to explain the basis for the differential targeting of gyrase over topoisomerase IV by zoliflodacin in *N. gonorrhoeae* cells. Zoliflodacin is a more potent inhibitor, induces higher levels of double-stranded and total DNA breaks, and establishes more stable cleavage complexes with *N. gonorrhoeae* gyrase compared with topoisomerase IV. Furthermore, it maintains higher levels of activity against gyrase that contains fluoroquinolone resistance mutations than resistant topoisomerase IV. These results agree with previous genetic studies in cultured *N. gonorrhoeae*, in which SPT-resistant cells harbored mutations in gyrase, but not topoisomerase IV.^46,60,81^

Several additional conclusions can be drawn from the present work. First, taken together with previous studies, zoliflodacin and other SPTs display activity against gyrase and topoisomerase IV from Gram-positive, Gram-negative, and atypical Gram-staining pathogens.^46,61,73,80^

Second, SPTs generate even higher levels of single-stranded than double-stranded DNA breaks with gyrase from some bacterial species. The induction of these single-stranded DNA breaks may further enhance the antibacterial activity of the SPT class.

Finally, as seen with fluoroquinolones, the primary targeting of gyrase could allow the stepwise evolution of highly resistant bacterial infections.^1,2,6,26^ Although zoliflodacin appears to be less mutagenic than fluoroquinolones,^60^ the fact that zoliflodacin also primarily targets gyrase suggests that target-mediated SPT resistance could eventually limit the clinical efficacy of this antibacterial class. The finding that H3D-005722 induces relatively higher levels of double-stranded DNA cleavage with topoisomerase IV than zoliflodacin indicates that it may be possible to develop new generations of SPTs that display more balanced cellular targeting of gyrase and topoisomerase IV. This is important because if a drug could kill bacterial cells through either gyrase or topoisomerase IV with similar efficacy, target-mediated resistance would require simultaneous mutations in both bacterial type II topoisomerases.^6,50,52,58,59^ Thus, balanced targeting of gyrase and topoisomerase IV could minimize the development of target-mediated resistance toward SPTs and extend the potential clinical lifespan of this antibacterial class.

## Materials and Methods

### DNA, Materials, and Enzymes

Negatively supercoiled pBR322 DNA was prepared from *E. coli* using a Plasmid Mega Kit (Qiagen) as described by the manufacturer. Relaxed pBR322 was generated by treating the negatively supercoiled plasmid with calf thymus topoisomerase I (Invitrogen) in 50 mM Tris-HCl (pH 7.5), 50 mM KCl, 10 mM MgCl_2_, 0.5 mM DTT, 0.1 mM EDTA, and 30 μg/mL bovine serum albumin (BSA) at 37 °C for 45 min followed by heat inactivation of topoisomerase I at 75 °C for 10 min.^82^ Positively supercoiled pBR322 DNA was prepared by treating 35 nM pBR322 (which is negatively supercoiled) with 420 nM recombinant *Archaeoglobus fulgidus* reverse gyrase as described previously.^72,83^ The number of positive supercoils generated by reverse gyrase was comparable with the number of negative supercoils in the original pBR322 preparations.^83^ Control reactions with negatively supercoiled plasmids omitted reverse gyrase but were otherwise subjected to treatment identical to that of the positively supercoiled molecules. Kinetoplast DNA (kDNA) was isolated from *Crithidia fasciculata* as described by Englund.^84^

Zoliflodacin (MedChemExpress) was stored at −20 °C as a 20 mM stock solution in 100% DMSO. H3D-005722 was synthesized using established methods as reported previously.^61^ This compound was stored at −20 °C as a 20 mM stock solution in 100% DMSO. All other chemicals were of analytical reagent grade.

All proteins were His-tagged. The identities of enzyme constructs were confirmed by DNA sequencing, and all enzymes were stored at −80 °C. In all assays, the stated enzyme concentration reflects that of the holoenzyme (A_2_B_2_).

Wild-type *N. gonorrhoeae* gyrase (GyrA, GyrB) and topoisomerase IV (ParC, ParE) subunits as well as mutant GyrA^S91F^ and GyrA^S91F/D95G^ gyrase were prepared as described previously.^34,46,63^*N. gonorrhoeae* mutant GyrA^D95G^ gyrase and mutant ParC^S87N^, ParC^E91G^, and ParC^S87N/E91G^ topoisomerase IV were generated using a QuickChange II XL site-directed mutagenesis kit (Agilent Technologies) with custom primers for the desired mutations. Mutant *N. gonorrhoeae* GyrA and ParC subunits were expressed and purified as described by Ashley et al.^70^ with the following modifications to optimize protein expression and lysis: (1) GyrA^D95G^ was expressed for 2.5 h and ParC^S87N^, ParC^E91G^, and ParC^S87N/E91G^ were expressed for 3 h before harvesting, and (2) cells were lysed by sonication using a digital sonifier. *N. gonorrhoeae* gyrase or topoisomerase IV was used as a 1:1 GyrA:GyrB or ParC:ParE mixture, respectively.

Wild-type *E. coli* gyrase (GyrA, GyrB) subunits were expressed and purified as described by Chan et al.,^63^ and *E. coli* wild-type topoisomerase IV (ParC, ParE, gift of Dr. Keir Neuman, NHLBI) subunits were expressed and purified as described by Peng and Marians.^14^*E. coli* gyrase or topoisomerase IV was used as a 1:1 GyrA:GyrB or ParC:ParE mixture, respectively.

Wild-type *B. anthracis* gyrase (GyrA, GyrB) and topoisomerase IV subunits (GrlA, GrlB) were expressed and purified as described by Dong et al.^85,86^*B. anthracis* gyrase or topoisomerase IV was used as a 1:1 GyrA:GyrB or GrlA:GrlB mixture, respectively.

### Gyrase-Catalyzed DNA Supercoiling

DNA supercoiling assays were based on a previously published protocol by Aldred et al.^87^ Reactions were performed in the absence of a compound or in the presence of increasing concentrations of zoliflodacin. Assays contained 15 nM wild-type or 25 nM mutant (GyrA^S91F^, GyrA^D95G^, or GyrA^S91F/D95G^) *N. gonorrhoeae* gyrase, 5 nM relaxed pBR322, and 1.5 mM ATP in a total volume of 20 μL of 50 mM Tris-HCl (pH 7.5), 175 mM KGlu, 5 mM MgCl_2_, and 50 μg/mL BSA. Assay mixtures were incubated at 37 °C for 20 min with wild-type and GyrA^D95G^, 25 min with GyrA^S91F/D95G^, or 30 min with GyrA^S91F^*N. gonorrhoeae* gyrase, which represents the minimum time required to completely supercoil the DNA in the absence of drug. Reactions were stopped by the addition of 3 μL of a mixture of 0.77% SDS and 77.5 mM Na_2_EDTA. Samples were mixed with 2 μL of loading dye [60% sucrose, 10 mM Tris-HCl (pH 7.9), 0.5% bromophenol blue, and 0.5% xylene cyanol FF] and incubated at 45 °C for 2 min before being subjected to electrophoresis on 1% agarose gels in 100 mM Tris-borate (pH 8.3) and 2 mM EDTA. Gels were stained with 1 μg/mL ethidium bromide for 20 min and then destained with distilled water for 10 min. DNA bands were visualized with medium-range ultraviolet light and quantified using an Alpha Innotech digital imaging system (Protein Simple). IC_50_ values (the concentration of drug required to inhibit enzyme activity by 50%) were calculated on GraphPad Prism using a nonlinear regression analysis with 95% confidence intervals.

### Topoisomerase IV-Catalyzed DNA Decatenation

DNA decatenation assays were based on previously published protocols by Anderson et al.^88^ and Aldred et al.^89^ Reactions were performed in the absence of compound or in the presence of increasing concentrations of zoliflodacin. Assays contained 20 nM wild-type *N. gonorrhoeae* topoisomerase IV, 5 nM kDNA, and 1 mM ATP in 20 μL of 40 mM HEPES-KOH (pH 7.6), 25 mM NaCl, 100 mM KGlu, and 10 mM Mg(OAc)_2_. Assay mixtures were incubated at 37 °C for 20 min, which represents the minimum time required to completely decatenate the kDNA in the absence of drug. Reactions were stopped, subjected to electrophoresis, and visualized as described for gyrase-catalyzed DNA supercoiling. IC_50_ values were calculated on GraphPad Prism using a nonlinear regression analysis with 95% confidence intervals.

### DNA Cleavage

DNA cleavage reactions were performed according to the procedure of Aldred et al.^89^ Reactions were performed in the absence of compound or in the presence of increasing concentrations of zoliflodacin or H3D-005722. For experiments performed with *N. gonorrhoeae* enzymes, assay mixtures contained 10 nM pBR322 and 100 nM wild-type, 100 nM GyrA^S91F^, 100 nM GyrA^D95G^, or 100 nM GyrA^S91F/D95G^ gyrase or 100 nM wild-type, 200 nM ParC^S87N^, 150 nM ParC^E91G^, or 150 nM ParC^S87N/E91G^ topoisomerase IV in a total volume of 20 μL of DNA cleavage buffer: 40 mM Tris-HCl (pH 7.9), 50 mM NaCl, 2.5% (w/v) glycerol, and 10 mM MgCl_2_. For reactions with *N. gonorrhoeae* topoisomerase IV that compared negatively and positively supercoiled DNA substrates, 200 nM enzyme was used to raise levels of baseline cleavage. Reactions were incubated at 37 °C for 30 min with wild-type and mutant (GyrA^S91F^, GyrA^D95G^, and GyrA^S91F/D95G^) *N. gonorrhoeae* gyrase, 20 min with mutant (ParC^S87N^, ParC^E91G^, and ParC^S87N/E91G^) *N. gonorrhoeae* topoisomerase IV, and 10 min with wild-type *N. gonorrhoeae* topoisomerase IV.

Assay mixtures for the other species contained 10 nM pBR322 and 500 nM wild-type *B. anthracis* gyrase, 100 nM wild-type *B. anthracis* topoisomerase IV, 100 nM wild-type *E. coli* gyrase, or 20 nM wild-type *E. coli* topoisomerase IV in a total volume of 20 μL of 40 mM Tris-HCl (pH 7.9), 50 mM NaCl, 2.5% (w/v) glycerol, and 5 or 10 mM MgCl_2_ for *E. coli* type II topoisomerases or *B. anthracis* topoisomerase IV, respectively, and 50 mM Tris-HCl (pH 7.5), 100 mM KGlu, 5 mM MgCl_2_, 50 μg/mL BSA, and 5 mM dithiothreitol (DTT) for *B. anthracis* gyrase. Reactions were incubated at 37 °C for 30 min with *B. anthracis* gyrase, 20 min with *E. coli* gyrase, and 10 min with topoisomerase IV from both species.

Enzyme-DNA cleavage complexes were trapped by adding 2 μL of 4% SDS followed by 2 μL of 250 mM EDTA (pH 8.0). Proteinase K was added (2 μL of a 0.8 mg/mL solution), and reaction mixtures were incubated at 45 °C for 30 min to digest the enzyme. Samples were mixed with 2 μL of loading buffer and heated for 2 min at 45 °C prior to electrophoresis in 1% agarose gels in 40 mM Tris-acetate (pH 8.3) and 2 mM EDTA containing 0.5 μg/mL ethidium bromide. DNA bands were visualized by midrange ultraviolet light and quantified using an Alpha Innotech digital imaging system (Protein Simple). Double-stranded DNA cleavage was monitored by the conversion of negatively supercoiled to linear plasmid molecules.

### Persistence of Gyrase/Topoisomerase IV-Cleaved DNA Complexes

The persistence of gyrase/topoisomerase IV-DNA cleavage complexes was determined as described previously by Aldred et al.^89^ Cleavage complexes were formed by combining 50 nM pBR322 and 100 nM *N. gonorrhoeae* gyrase or 200 nM *N. gonorrhoeae* topoisomerase IV in the presence of 100 μM zoliflodacin in a total volume of 20 μL of *N. gonorrhoeae* gyrase/topoisomerase IV-DNA cleavage buffer. Parallel control experiments were conducted to assess cleavage complexes formed in the absence of the SPT by combining 50 nM pBR322 and 500 nM *N. gonorrhoeae* gyrase or topoisomerase IV in 20 μL of DNA cleavage buffer. Reactions were incubated at 37 °C until DNA cleavage/ligation equilibria were reached (30 min with gyrase and 10 min with topoisomerase IV) and diluted 20-fold in DNA cleavage buffer lacking Mg^2+^. Reactions (20 μL samples) were stopped at time points ranging from 0 to 60 min, subjected to electrophoresis, and visualized as described above for DNA cleavage. Linear DNA cleavage products at time zero were set to 100% to allow direct comparison between different conditions, and the persistence of cleavage complexes was determined by the decay of linear (double-stranded breaks) reaction products over time. Cleavage complex stability (half-life, *t*_1/2_) was calculated on GraphPad Prism using a nonlinear regression analysis with 95% confidence intervals.

### Gyrase/Topoisomerase IV-Mediated DNA Ligation

DNA ligation mediated by *N. gonorrhoeae* gyrase and topoisomerase IV was monitored using the procedure of Aldred et al.^89^ Initial reactions contained 10 nM pBR322 and 100 nM wild-type *N. gonorrhoeae* gyrase or topoisomerase IV in the absence or presence of 100 μM zoliflodacin. DNA cleavage–ligation equilibria were established for 30 min with gyrase and 10 min with topoisomerase IV at 37 °C. DNA ligation was initiated by shifting samples from 37 to 65 °C, which allows enzyme-mediated ligation but prevents new rounds of DNA cleavage from occurring.^69^ This results in a unidirectional sealing of the cleaved DNA. Reactions were stopped at time points ranging from 0 to 60 s, subjected to electrophoresis, and visualized as described above for DNA cleavage. Linear DNA cleavage products at time zero were set to 100% to allow direct comparison between different conditions, and DNA ligation of double-stranded breaks was monitored by the loss of linear DNA. The rate of DNA ligation (*t*_1/2_) was calculated on GraphPad Prism using a nonlinear regression analysis with 95% confidence intervals.
